# The price of neonatal intensive care outcomes – in-hospital costs of morbidities related to preterm birth

**DOI:** 10.3389/fped.2023.1068367

**Published:** 2023-02-07

**Authors:** Asaph Rolnitsky, Sharon Unger, David Urbach, Chaim M. Bell

**Affiliations:** ^1^ Department of Paediatrics, University of Toronto, Toronto, ON, Canada; ^2^ Department of Surgery, University of Toronto, Toronto, ON, Canada; ^3^ Department of Medicine, University of Toronto, Toronto, ON, Canada

**Keywords:** neonatology, cost, healthcare services, prematurity, NICU

## Abstract

**Background:**

Neonatal care for preterm babies is prolonged and expensive. Our aim was to analyze and report costs associated with common preterm diagnoses during NICU stay.

**Methods:**

We analyzed data from the Ontario healthcare data service. Diagnoses were collated by discharge ICD codes, and categorized by gestational age. We calculated typical non parametric statistics, and for each diagnosis we calculated median shifts and generalized linear mode.

**Results:**

We included data on 12,660 infants between 23 and 30 weeks gestation in 2005-2017. Calculated cost increment with diagnosis were: Intestinal obstruction: $94,738.08 (95%CI: $70,093.3, $117,294.2), Ventriculoperitoneal shunt: $86,456.60 (95%CI: $60,773.7, $111,552.2), Chronic Lung Disease $77,497.70 (95%CI: $74,937.2, $80,012.8), Intestinal perforation $57,997.15 (95%CI:$45,324.7, $70,652.6), Retinopathy of Prematurity: $55,761.80 (95%CI: $53,916.2, $57,620.1), Patent Ductus Arteriosus $53,453.70 (95%CI: $51,206.9, $55692.7, Post-haemorrhagic ventriculomegaly $41,822.50 (95%CI: $34,590.4, $48,872.4), Necrotizing Enterocolitis $39,785 (95%CI: $35,728.9, $43,879), Meningitis $38,871.85 (95%CI: $25,272.7, $52,224.4), Late onset sepsis $32,954.20 (95%CI: $30,403.7, 35.515), Feeding difficulties $24,820.90 (95%CI: $22,553.3, $27,064.7), Pneumonia $23,781.70 (95%CI: $18,623.8, $28,881.6), Grade >2 Intraventricular Haemorrhage $14,777.38 (95%CI: $9,821.7, $20,085.2). Adjusted generalized linear model of diagnoses as coefficients for cost confirmed significance and robustness of the model.

**Conclusion:**

Cost of care for preterm infant is expensive, and significantly increases with prematurity complication. Interventions to reduce those complications may enable resource allocation and better understanding of the needs of the neonatal health services.

## Introduction

Preterm birth affects up to ten percent of births world-wide (1). Preterm babies, defined as those born before 37 weeks gestation, are often admitted to neonatal intensive care units (NICUs), where highly specialized medical care is provided until hospital discharge. The standard stay in NICU is prolonged and for very preterm infants typically includes multiple ventilatory interventions, diagnostic imaging, and invasive procedures. Diagnoses made in the NICU and neonatal outcomes are important predictors of life-long chronic medical conditions and impaired quality of life (2). The routinely reported neonatal outcomes include intraventricular haemorrhage, sepsis, chronic lung disease, necrotizing enterocolitis, retinopathy of prematurity, and meningitis. Indeed, a typical list of neonatal diagnoses at NICU discharge is an important predictor for long term outcome (3, 4). Over time, survival of the most preterm infants has improved consistently (5–7). Today, in many units survival of preterm infants, born before 26 weeks of gestation is higher than 80% and normal growth and development is reported in most of early prematurity survivors, with a better prognosis for those who had a more stable, morbidity-free NICU stay (8).

Costs for NICU admissions have been of interest ever since the concept of specialized neonatal units emerged (9, 10). The advanced technological support enables the survival of younger and more fragile infants, but this requires lengthy hospitalizations and large medical teams (11, 12). Medical actions, such as surgical procedures, prolonged ventilation, and parenteral nutrition also contribute to improved outcomes. The complex care results in increased costs for hospital resources, equipment, and staffing. Recent studies (13, 14) note that NICU patients were amongst the highest consumers of hospital resources, despite comprising a small fraction of hospital patients. The overall cost of NICU care has now become a source of interest to policy makers, clinicians, hospital administrators, and the public. Numerous publications estimating the cost of NICU care have demonstrated high costs (15, 16), but also cost-effectiveness (17–21), and clear cost-utility even at the most preterm, peri-viable 23–25 weeks of gestational age at birth (16, 20). Several publications have estimated the cost of NICU (22) according to different payor perspectives with extensive regional variation (23, 24). Indeed, most work has adopted a broad perspective to costs and diagnoses. However, a more detailed analysis of NICU costs can be helpful in delineating the highest cost components of the total NICU stay and specific diagnoses. Such an analysis can be important when considering resource allocation as well as when estimating changing needs. Thus, we embarked on analyzing the various costs associated with important neonatal diagnoses accrued in all NICUs in Ontario, Canada.

## Methods

Data was analysed on cost of hospital stay using data held at the Institute for Clinical Evaluative Services (ICES), in Ontario, Canada (25). ICES is a provincial healthcare research institute that is entrusted with the provincial medical services information. This includes costs, diagnoses, and demographic information (26). ICES data is subject to quality checks, with ≥98% correlation with patient charts in multiple studies (27–28).

All preterm livebirths at 23–30 weeks gestational age in Ontario, Canada from 2010 to 2017 were included. This includes years when resuscitation of 23-week infants was increasingly supported in NICUs across the province. Delays in data availability due to extensive quality and audit checks precluded utilizing more recent information.

Costs included patient-level cost of hospital care from birth until discharge from hospital or death, including physicians' compensation and in hospital services. ICES costing methodology (29) is based on person-level costing by utilization of services. For our analysis, the cost included the totals reported at the government level, thus reflecting the complete cost to the payor - the Ontario Ministry of Health and Long-Term Care - thereby providing a public perspective, without birth location, or level or care status. The costs were adjusted to the yearly published Canadian Healthcare Consumer Price Index (30) to ensure comparability between years.

We sorted the most frequently diagnosed conditions in the diagnoses list, and included the clinically important neonatal diagnoses, as reported by the Vermont-Oxford Network (31), Canadian Neonatal Network, and the International Neonatal Network (8). Diagnoses of “preterm infant” or “very low birth weight”, were not included for analysis as these were the inclusion criteria. Where appropriate, we collated similar diagnoses, narrowing the list to 15 diagnoses based on clinical importance. For example, all late onset infections were recoded as one diagnostic outcome- “late-onset sepsis” but did not include other infections such as meningitis. All diagnoses related to long term lung disease were recoded as “chronic lung disease”.

The study was approved by Sunnybrook Hospital Research Ethics Board and ICES.

### Statistical analysis

We calculated means, 95% confidence intervals [95%CI], medians, interquartile ranges [IQR] and standard deviations (SD) for each diagnosis. We tested the distributions of the costs to select appropriate statistical tests and for proper model fitting. We limited the data to that of infants who survived more than 3 days for the cost analyses as those who died earlier were typically extremely sick or received palliative care and would not have the diagnoses of interest. We then evaluated costs for each diagnosis independently, comparing the cost of stay with vs. without the specific diagnosis. For the analysis of geometrically distributed data, we used the Wilcoxon-rank-sum test. After comparison of each diagnosis's cost, we confirmed the findings by constructing a gamma-fitted, generalized linear model to estimate the cost coefficient associated with each diagnosis to the total cost model after removal of the largest points of leverage from the model using Cook's distance. This technique isolates each diagnosis and is useful in conditions that can be continuous (such as intraventricular haemorrhage that progresses to ventriculomegaly). Coefficients for the model were Retinopathy of Prematurity, Patent Ductus Arteriosus, Feeding difficulties, Necrotizing Enterocolitis, Ventriculomegaly, Intestinal perforation, Ventriculoperitoneal Shunt, Acute Kidney Injury, non-NEC colitis, Severe Intraventricular Haemorrhage, Intestinal Obstruction, Late Onset Sepsis, Chronic Lung Disease, as well as IUGR, gestational age, survival >3 days and multiple pregnancy. Model robustness was assessed by pseudo-R-square, and the diagnoses coefficients were exponentiated for reporting. We performed the analyses using R statistical language, v. 3.6.5.

## Results

We analyzed 12,660 cost records from 2010 to 2017. This included 626 infants born at 23 weeks, 897 at 24 weeks, 1,130 at 25 weeks, 1,364 at 26 weeks, 1,559 at 27 weeks, 1,830 at 28 weeks, 2,253 at 29 weeks, and 3,001 infants at 30 weeks. The overall survival of the cohort beyond 3 days was 90.47% (11,454 infants). The median length of NICU stay was 42 days (IQR = 29–64) (Table 1).

**Table 1:** Cost data review.

	*n*	min	max	median	IQR	mean	SD
Gestational Age (weeks)		23	30	28	26–29	27.52	2.15
Birth weight (gr)		276	4770	1,090	820–1375	1142	463.4
Total cost ($CAD)		400.6	2,057,072	77,132.9	48,338–126,680	98270.3	92,971.3
Total length of stay to discharge/death (d)		1	676	42	29–64	49.96	38.08
Daily cost ($CAD)		14	64,967	1,935	1,435–2,586	2,149	1,437.84
Survived 3 days	11,454					90.5%	
Multiples	3,504					27.7%	
Intrauterine growth restriction	1135					8.97%	

The 15 most frequent diagnoses and their rate of occurrence among survivors more than 3 days are presented in Table 2. 58% of infants had more than one diagnosis, 34% of patients had more than two diagnoses, and 1.7% had more than five diagnoses.

**Table 2:** Frequency and median costs of NICU care, by diagnosis.

Diagnosis	*n*	%	Wilcoxon Median Shift	95% CI
Intestinal obstruction	44	0.38%	$94,738.08	$70,093.3, $117,294.2
Ventriculoperitoneal shunt (VPS)	36	0.31%	$86,456.60	$60,773.7, $111,552.2
Chronic Lung Disease (CLD)	2941	25.68%	$77,497.70	$74,937.2, $80,012.8
Intestinal perforation (Perf)	267	2.33%	$57,997.15	$45,324.7, $70,652.6
Retinopathy of Prematurity (ROP)	5333	46.56%	$55,761.80	$53,916.2, $57,620.1
Patent Ductus Arteriosus (PDA)	4212	36.77%	$53,453.70	$51,206.9, $55692.7
Post-haemorrhagic ventriculomegaly (VM)	346	3.02%	$41,822.50	$34,590.4, $48,872.4
Necrotizing Enterocolitis (NEC)	1244	10.86%	$39,785	$35,728.9, $43,879
Meningitis	96	0.84%	$38,871.85	$25,272.7, $52,224.4
Late onset sepsis	3226	28.16%	$32,954.20	$30,403.7, 35.515
Feeding difficulties	3805	33.22%	$24,820.90	$22,553.3, $27,064.7
Pneumonia	623	5.44%	$23,781.70	$18,623.8, $28,881.6
Grade >2 Intraventricular Haemorrhage (sIVH)	650	5.67%	$14,777.38	$9,821.7, $20,085.2
Acute Kidney Injury (AKI)	189	1.65%	$9,085.50	$−1281.2, $19,029
non-NEC Colitis (Colitis)	120	1.05%	$2,867.90	$−5,511.5$, 12,225.6

The top 5 diagnoses and the frequency in our cohort were: retinopathy of prematurity (ROP, 46.56%), Patent ductus arteriosus (PDA, in 36.77% of the infants), feeding difficulties (feeding, 33.22%), late onset sepsis (28.16%), and chronic lung disease (CLD, 25.68%). Notably, 27.67% were multiple pregnancies and 9% had a diagnosis of intrauterine growth restriction (IUGR).

**Figure 1:**
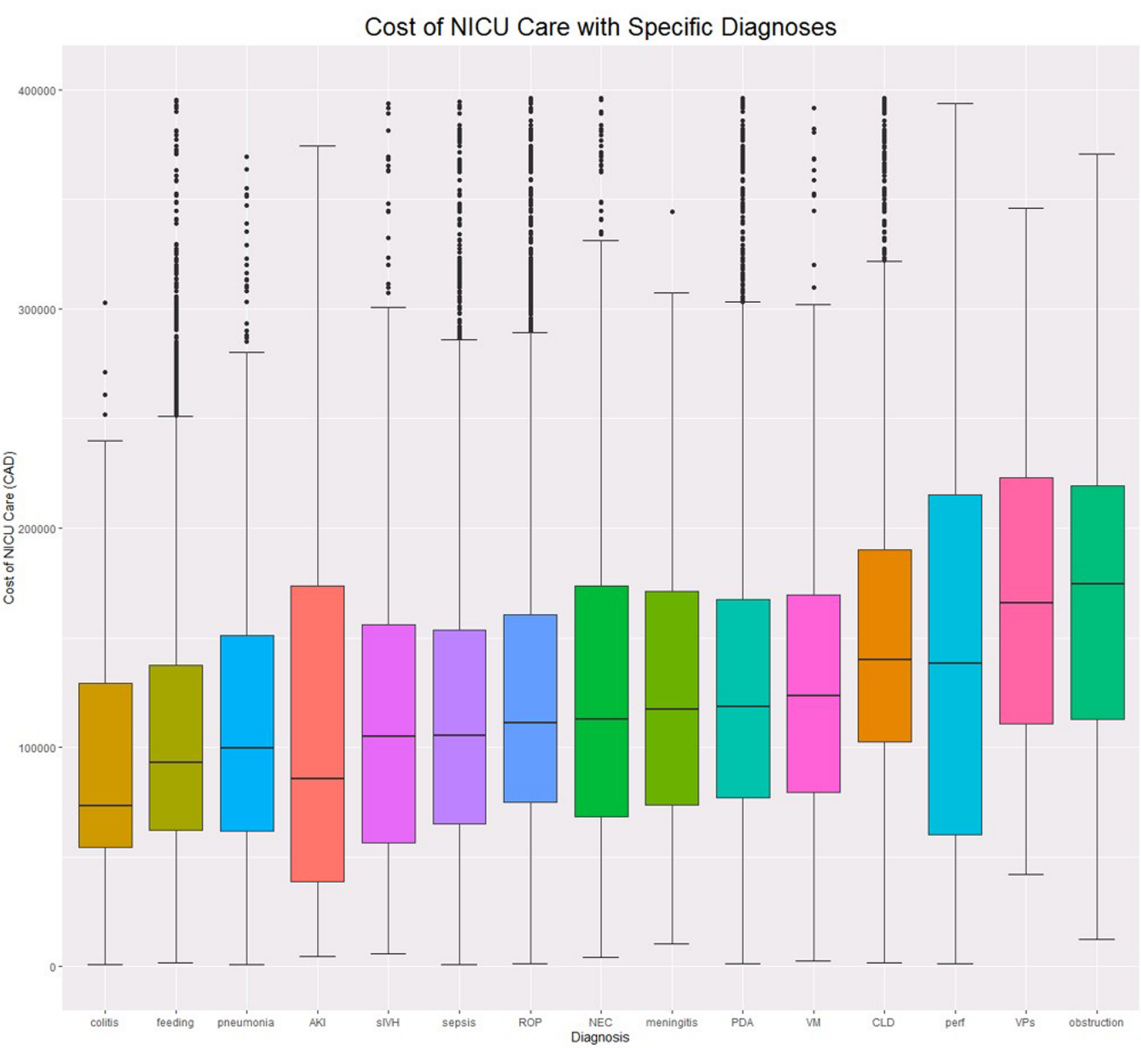
Cost boxplots by diagnosis. ROP, Retinopathy of prematurity; PDA, Patent Ductus Arteriosus; feeding, Feeding difficulties; NEC, Necrotizing Enterocolitis; VM, Ventriculomegaly; perf, Intestinal perforation; VPs, Ventriculoperitoneal Shunt; AKI, Acute Kidney Injury; colitis, non-NEC colitis; sIVH, Severe Intraventricular Haemorrhage; obstruction, Intestinal Obstruction; sepsis, Late Onset Sepsis; CLD, Chronic Lung Disease.

The median cost was $77,132.90 (IQR = $48,338-$126,680) per NICU admission. Among infants who survived more than 3 days, the median cost was $84,774 (IQR = $55,797-$133,594). Figure 1 demonstrates cost distributions by diagnosis.

We evaluated incremental costs associated with the top 15 diagnoses. (Table 2). The 5 diagnoses that were associated with the highest incremental costs were: intestinal obstruction $94,738.08, (95%CI: $70,093.3, $117,294.2), ventriculo-peritoneal (VP) shunt $86,456.60, (95%CI: $60,773.7, $111,552.2), Chronic Lung Disease (CLD) $77,497.70, (95%CI: $74,937.2, $80,012.8), intestinal perforation $57,997.15, (95%CI: $45,324.7, $70,652.6), Retinopathy of Prematurity (ROP) $55,761.80, (95%CI: $53,916.2, $57,620.1). Table 3 details length of stay increases associated with each diagnosis. Longer list of diagnoses was associated with incremental median cost increase, up to 8 diagnoses (fitted model, *R*^2 ^= 0.9, *p *<< 0.001).

**Table 3:** Median shift of length of stay for each diagnosis.

Diagnosis	Wilcoxon Median Shift (days)	95% CI
Intestinal obstruction	22	9, 35
Ventriculoperitoneal shunt (VPS)	42	29, 59
Chronic Lung Disease (CLD)	38	37, 39
Intestinal perforation (Perf)	21	14, 27
Retinopathy of Prematurity (ROP)	25	24, 26
Patent Ductus Arteriosus (PDA)	23	22, 24
Post-haemorrhagic ventriculomegaly (VM)	13	10, 17
Necrotizing Enterocolitis (NEC)	12	10, 14
Meningitis	12	6, 20
Late onset sepsis	16	15, 17
Feeding difficulties	8	7, 10
Pneumonia	14	11, 17
Grade >2 Intraventricular Haemorrhage (sIVH)	7	4, 9
Acute Kidney Injury (AKI)	14	8, 20
non-NEC Colitis (Colitis)	2	−3,6

We used a fitted generalized linear model to further assess the diagnoses' contribution to cost after adjusting for various covariates and isolating each diagnosis. The calculated costing coefficients provide the effect of each diagnosis on the total cost and are presented in Table 4. The top 5 contributors to increased cost were: VP shunt 1.747, (95%CI: 1.45–2.11), intestinal obstruction 1.585, (95%CI:1.38–1.82), CLD 1.333, (95%CI: 1.31–1.36), ROP 1.24, (95%CI: 1.22–1.26), PDA 1.143, (95%CI: 1.13–1.16). The diagnoses of acute kidney injury, colitis, pneumonia, meningitis, and severe intraventricular haemorrhage were all nonsignificant (*P* > 0.01). The model robustness was confirmed with a pseudo-R-squared = 0.75.

**Table 4:** Generalized linear cost model adjusted for gestational age, survival >72 h, IUGR and multiple pregnancy. See also Figure 2.

	Diagnosis	Exponentiated Coefficient	95% Confidence Interval
1	Ventriculoperitoneal Shunt (VPS)	1.747	1.45–2.11
2	Intestinal Obstruction	1.585	1.38–1.82
3	Chronic Lung Disease (CLD)	1.333	1.31–1.36
4	Retinopathy of Prematurity (ROP)	1.24	1.22–1.26
5	Patent Ductus Arteriosus (PDA)	1.143	1.13–1.16
6	Intestinal perforation (Perf)	1.125	1.07–1.19
7	Necrotizing Enterocolitis (NEC)	1.117	1.09–1.15
8	Meningitis	1.098	1–1.2
9	Ventriculomegaly (VM)	1.081	1.04–1.13
10	Feeding difficulties	1.078	1.06–1.1
11	Late Onset Sepsis	1.073	1.06–1.09
12	Non-NEC Colitis (Colitis)	1.016	0.94–1.09
13	Pneumonia	1.003	0.97–1.04
14	Severe Intraventricular Haemorrhage (sIVH)	0.956	0.93–0.99
15	Acute Kidney Injury (AKI)	0.934	0.87–1

**Figure 2:**
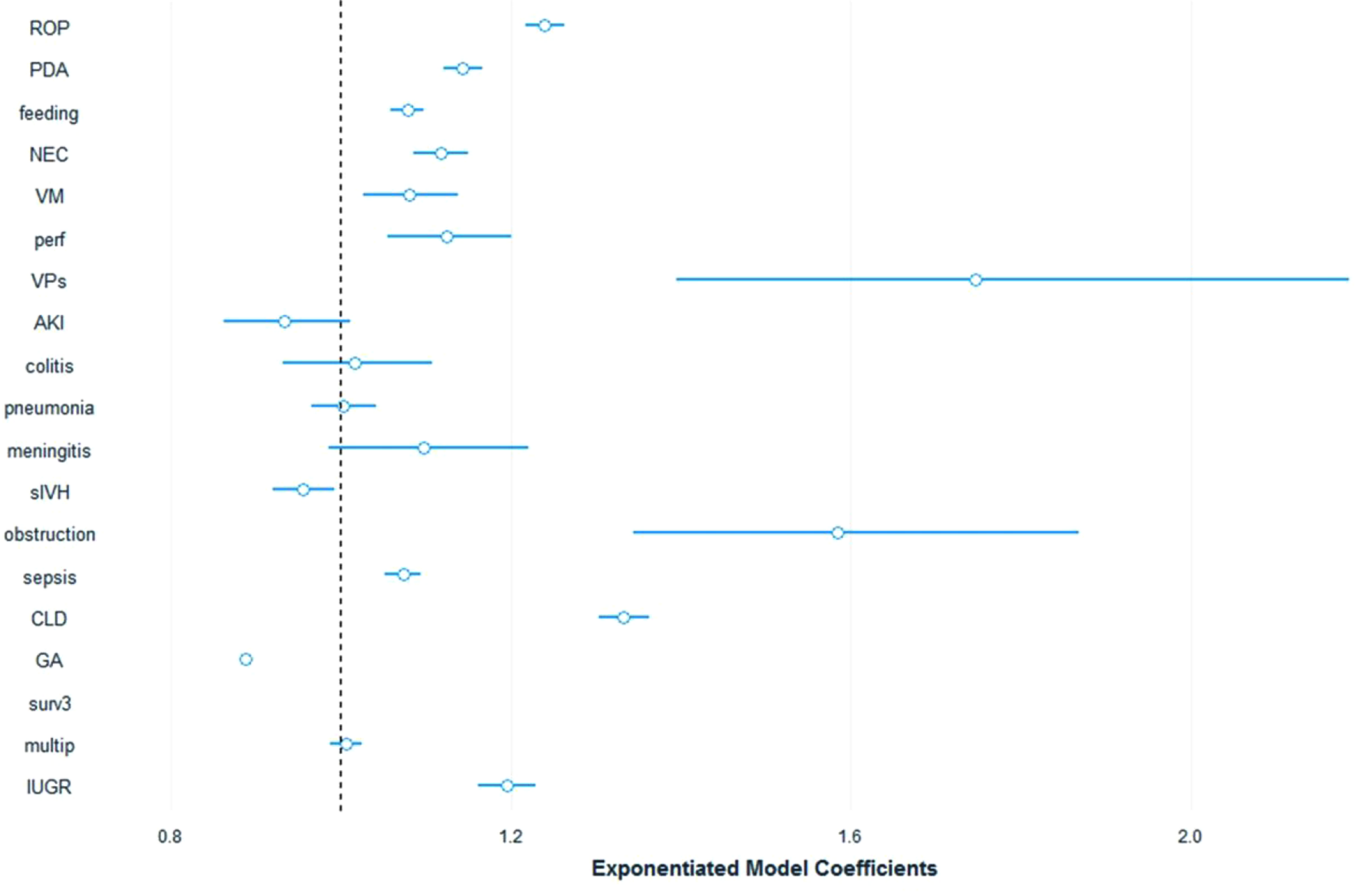
Cost model coeffecients plot. ROP, Retinopathy of prematurity; PDA, Patent Ductus Arteriosus; feeding, Feeding difficulties; NEC, Necrotizing Enterocolitis; VM, Ventriculomegaly; perf, Intestinal perforation; VPs, Ventriculoperitoneal Shunt; AKI, Acute Kidney Injury; colitis, non-NEC colitis; sIVH, Severe Intraventricular Haemorrhage; obstruction, Intestinal Obstruction; sepsis, Late Onset Sepsis; CLD, Chronic Lung Disease.

## Discussion

We analyzed the costs of neonatal diagnoses in a large cohort of preterm infants, born at less than or equal to 30 weeks gestational age, using a validated, population-level costing database. This study continues the analysis of NICU costing by age (24) and by region (23). We found that the median cost of care for the entire cohort of 12,600 infants was $77,132.9. Among infants who survived more than 3 days, the median cost was $84,773.8. The most relevant diagnoses for this cohort were common, and median costs related to each of the top five diagnoses were: intestinal obstruction $94,738.08, VP shunt $86,456.6, CLD $77,497.7, intestinal perforation $57,997.15, and any-stage ROP $55,761.8.

Our data confirms the overall high cost of neonatal care. As well, we note that significant increases in costs can be attributed to neonatal morbidities which occur commonly and when occur as compound morbidities. These findings are confirmed by the robust model that adjusted for several covariates. While our analysis did not show significant cost change for severe IVH or renal injury at the model level, it did confirm cost changes in the nonparametric tests for the severe IVH. We hypothesize that the expectant management of IVH and renal injury did not change the cost of care for those infants who did not develop secondary complications. The cost data also showed that some diagnoses had up to 10% cost outliers, which fits the distributions typical of costing data. Our Generalized linear model mitigated the effect of the outlier, as demonstrated in previous studies (32).

The general costs estimated are close to those determined in one comparable study (22), and higher than those found in a different, less recent, comparable study (33). The former (Rios et al.) (22) detailed the total costs by gestational age using a costing predictive model based on level 3 NICUs only and did not include specific diagnoses as outcomes. The latter used a predictive model based on data obtained before 2007 and included fewer extremely preterm infants. Further, the findings were influenced by a far higher mortality rate (56%). To our knowledge, this is the first report of detailed additional costs related to various diagnoses, and not by gestational age, thus answering questions about the financial implication of a specific neonatal morbidity, regardless of a specific age.

Cost analyses for specific medical conditions can be challenging and depend on the study perspective, the scope of the problem, and the level of analysis (34–37). In the absence of an established cost database, studying the specific “price tag” of a condition usually requires a complex methodology (35, 38) and can have limited applicability (39) or validity (40). Nevertheless, there is an increasing interest in costing studies, and specifically in neonatal health services analyses that started with the establishment of neonatal intensive care (9, 10). Studying cost of various medical conditions can provide knowledge of resource requirements, improvement targets, system performances, and inequities (34, 37, 41), thus prompting policy evaluation and potentially reducing costs (42–46). Costing of medical complication can aid targeting improvement efforts in reducing complications life infections or chronic lung disease, or allocation support for services for infants with some of the complicaitonsThis knowledge can also serve as a basis for future cost-effectiveness and cost-utility analyses (47). While there is a debate on the effect of cost studies on policymaking (39, 43–45), knowledge about cost can be one tool for policy adjustment and improved resource allocation. Financial decision regarding neonatal services can use this data for future planning with the increase survival of infants and the increase in preterm birth. Similar attempts were made for dementia care (48), diabetes (49), and cardiovascular disesase (50).

Our study has important strengths. First, we analyzed a large, population-based cohort of 12,660 births, representing all preterm infants less than 30 weeks of gestational age in a jurisdiction with over 14 million people. These infants were cared for in any level of NICU and we included years during which support for 23-week gestational age infants was frequently provided. Thus, the costs of recent practices to support the most preterm infants are included.

Second, we stratified the cost data to a standard year, using the Canadian healthcare consumer price index to ensure comparability between years. Notably, this has changed little since that time. Third, we employed a Ministry of Health perspective for costing, acting as the payor, which includes all the costs incurred by the infants during their hospital stay. This represents real-life, bottom-line expenditures and is meaningful to the decision makers and the public. Fourth, we sorted the most common and important diagnoses in neonatal care and recoded them to be clinically meaningful to the care providers. Finally, we constructed a robust costing model to reliably demonstrate the cost coefficients and cost increments associated with the diagnoses of preterm infants.

Our study also has some limitations. We were provided ICD-10 diagnoses that are, like every abstractor-dependent coding process, prone to potential errors. Most of the preterm-related diagnoses are, however, quite specific, and less likely to be miscoded. An example here is Retinopathy of prematurity that was not subcategorized to stages and thus the costing of the higher stages, that are associated with longer stay, interventions, and higher cost, are not differentiated here. Second, the data is from one province only, Ontario, Canada. Ontario has the largest population in Canada with over 14 million people (more than a third of the country) and the largest number of preterm births. Thus, it can be seen as a representative national sample population with applicability to other countries. Moreover, the analysis is population-based so there is little bias from missed information on infants. Third, the study collected data until 2017, as the finalized, validated, coded data lagged study inception. The completeness of the data, its high quality, and the relative consistency in infant care enable a valid use of its results. Fourth, this analysis does not include other expenses for families related to patient care. It also does not include any healthcare costs following discharge from the initial NICU hospitalization. These elements were beyond the scope of this study as we only examined costs of hospital stay from a public perspective. Further research can elucidate the concepts of family-related expenditures and ensuing healthcare services. Fifth, the study cannot sub-analyze costs at program or care levels or cost inputs as it uses the final costs per patient, regardless of transfers between unit. This information can be important for comparison between units and NICU levels of care but was beyond of the scope of this study and could not determine where each diagnosis occurred. Last, we did not perform a formal cost-utility analysis. This would involve a more detailed assessment of infant and family quality of life that was beyond the scope of our work. Previous analyses have clearly demonstrated that even at the lowest gestational age, NICU care is considered to demonstrate cost-effectiveness with estimates well below commonly employed cost-utility thresholds (16, 20, 51). Additional considerations regarding the value of NICU care is an ethical discussion that has also been studied previously (52).

Our study collected costing data at the payor level of extremely preterm babies and demonstrated a high cost of NICU stay, and significant costs associated with morbidities that are common in preterm born infants. Understanding these costs will enable better resource allocation and funding consideration for this fragile population.

## Data Availability

The original contributions presented in the study are included in the article/Supplementary Material, further inquiries can be directed to the corresponding author/s.
